# Validating GenAI feedback in suicide prevention training: a mixed-methods study of QPR skill assessment

**DOI:** 10.3389/fmed.2025.1709743

**Published:** 2026-01-15

**Authors:** Yuval Haber, Yossi Levi-Belz, Yael Segal Elbak, Zohar Elyoseph, Inbar Levkovich

**Affiliations:** 1 The Program for Hermeneutics and Cultural Studies, Interdisciplinary Studies Unit, Bar-Ilan University, Ramat Gan, Israel; 2 The Lior Tsfaty Center for Suicide and Mental Pain Studies, University of Haifa Mount Carmel, Haifa, Israel; 3 The School of Therapy Counseling and Human Development, Faculty of Education, University of Haifa, Haifa, Israel; 4 Faculty of Education, Tel Hai College, Upper Galilee, Israel

**Keywords:** suicide prevention, gatekeeper training, artificial intelligence, QPR model, medical simulation, mental health training, conversation analysis, AI-generated feedback

## Abstract

**Background:**

Gatekeeper training using the Question, Persuade, and Refer (QPR) model has become a key strategy in suicide prevention. Yet traditional QPR-based training methods are limited by their lack of scales, interactive practice and reliable assessments of skill acquisition. Generative AI (GenAI)-driven simulators can provide a novel solution for this critical gap by offering scalable and cost-effective practices that can be culturally adapted, thus democratizing access to skills training without the potential embarrassment of live roleplay. Nevertheless, the rigorous validation of AI systems as reliable evaluation tools remains an open question. This study seeks to validate the reliability of AI-based assessments in the high-stakes context of suicide prevention, thus constituting a critical step toward using GenAI for scalable skill evaluation.

**Methods:**

Three independent experts rated 54 simulated QPR conversations to establish the empirical reliability of a GenAI simulator feedback in the context of suicide prevention training. The primary analysis compared the automated numerical scores of the AI feedback against this human benchmark (RQ1). Secondary analyses included examination of the influence of participant characteristics on ratings (RQ2) and qualitative assessment of the AI feedback for pedagogical depth and accuracy across varied performance levels (RQ3).

**Results:**

The primary finding (RQ1) exhibited a moderate-to-strong positive correlation (*r* = 0.519–0.776) between the GenAI adherence scores and the human-rated benchmark, providing initial evidence for the tool’s reliability. No significant gender-based differences were found in either GenAI or human ratings, supporting the study’s aim for an unbiased tool (RQ2). Qualitative analysis demonstrated GenAI’s ability to accurately identify key QPR components and deliver nuanced in-depth feedback (RQ3).

**Conclusion:**

This study provides critical initial evidence that GenAI can serve as a reliable feedback tool for evaluating complex crisis intervention skills. Its ability to provide consistent, scalable, and unbiased assessments opens new possibilities for accessible evidence-based training. Despite these strong foundational capabilities, the findings also highlight the need for further calibration to align GenAI’s judgment more closely with expert human nuances and enable it to evolve beyond a purely performance-focused tool.

## Introduction

Suicide is a major public health challenge worldwide and a leading cause of preventable deaths (1), with profound psychological and economic consequences for individuals, families, and healthcare systems (2). The widespread and unpredictable nature of suicide highlights the urgent need for scalable and evidence-based prevention strategies. Gatekeeper training has emerged as a cornerstone of such efforts. It aims to equip individuals in frontline roles (e.g., educators, healthcare providers, and community leaders) with the skills to recognize warning signs, engage in supportive conversations, and refer at-risk individuals to appropriate services (3–5). In addition to skill building, gatekeeper programs help reduce stigmas and normalize mental health support (6, 7).

The Question, Persuade, and Refer (QPR) model is one of the most widely implemented and empirically supported training models (3, 8, 9). QPR offers a three-step approach: asking directly about suicidal ideation, persuading the individual to seek help, and referring them to appropriate support services (10). The model’s clarity and adaptability make it well suited to diverse contexts, including schools, universities, healthcare settings, and indigenous communities (5, 11, 12).

Despite its accessibility and proven impact, traditional QPR training has several important and inherent limitations. These include reliance on didactic instruction, limited opportunities for active rehearsal, and the logistical challenges of in-person role-play (11, 13). In most group training settings, only a few participants engage directly in simulations, while others observe them passively, resulting in unequal experiential learning (4, 12). Evaluation methods often depend on self-reported confidence or hypothetical case discussions, which provide limited insight into participants’ real-time application of crisis intervention skills (14, 15). These limitations underscore the critical need for tools that support both skill development and valid behavior-based assessment.

Generative Artificial Intelligence (GenAI) powered by large-language models offers a novel and promising solution to these challenges. Recent studies have demonstrated GenAI’s potential in mental health contexts (36), including suicide risk screening (16), diagnostic decision support (17), therapeutic self-exploration (18), and clinical training (19). GenAI-based simulations can generate interactive and psychologically rich environments that simulate real-world crisis encounters. These environments enable participants to practice complex interpersonal skills using emotionally responsive virtual characters, receive real-time feedback, and repeat the experience as required (7, 20, 21).

Unlike traditional role-playing in which only a few individuals engage at a time, GenAI simulations democratize participation, allowing every user to participate actively and receive immediate personalized responses (11, 22). These simulations collected granular data on language use, emotional tone, and adherence to core QPR components, thus generating structured, pedagogically meaningful feedback (19, 22). Recent research has found that participants in GenAI-based training report increased confidence, greater emotional realism, and enhanced self-reflection in comparison to those undergoing conventional training (18, 22).

Two recent studies specifically evaluated the GenAI-based simulator used in the present study. In both studies (19, 22), mental health professionals and gatekeepers engaged in real-time text-based conversations with an emotionally responsive AI character that simulated an individual in the midst of a suicidal crisis. The quantitative results showed a significant increase in post-intervention self-efficacy, while the qualitative findings emphasized the simulator’s realism and pedagogical value. Participants reported improvements in their intervention skills and personal insight, while noting the importance of maintaining human connections in mental health training. These findings demonstrate the tool’s promise in skill acquisition and informed reflection. Yet, participants also raised concerns about maintaining emotional nuances, relational authenticity, and the need to ensure trauma-informed design in digital learning environments (6, 18).

## The current study

Despite the promise of GenAI in suicide prevention training, the validity and fairness of its feedback remain open questions. Communication styles, such as expressiveness, tone, and directness, can vary by gender, culture, or personality, potentially influencing how humans and GenAI assess conversations (23). Without adequate safeguards, GenAI tools trained on biased or limited datasets risk perpetuating inequities in the evaluation (37, 38). For GenAI to be a trusted evaluator in high-stakes domains such as suicide prevention, its feedback must be accurate, unbiased, and aligned with expert human judgment (17, 24).

This study aimed to evaluate the reliability, fairness, and pedagogical validity of GenAI feedback in QPR gatekeeper training. Rather than relying on hypothetical cases or retrospective self-assessments, the study analyzed actual text-based interactions between participants and a GenAI-simulated individual experiencing suicidal ideation. Our main question was to determine the extent to which GenAI-generated feedback can serve as a valid and pedagogically meaningful tool for evaluating the real-time application of QPR skills in suicide prevention training. Specifically, we sought to determine whether GenAI adherence scores aligned with expert human ratings (RQ1), whether participant characteristics or interaction features influenced these evaluations (RQ2), and to what extent GenAI’s narrative feedback reflected the accuracy, depth, and tone necessary for formative training (RQ3). By examining real-time interactions from a live training context, this study aimed to move beyond theoretical speculation and simulation design, instead providing empirical evidence for the role of GenAI in assessing and shaping critical gatekeepers’ competencies.

## Method

### Sample

The dataset included 54 simulated conversations that were evaluated for adherence to the QPR model. While acknowledging this modest sample size, this initial study was designed as a proof of concept, adopting a cautious approach to AI integration in the sensitive domain of suicide prevention training. The conversation participants included 30 men, 20 women, and four individuals whose gender could not be identified. Additional sociodemographic data (e.g., age and profession) were collected via an optional, anonymous survey to protect participant privacy, in line with ethical guidelines. The characteristics presented below reflect a subset of N = 48 participants who voluntarily completed both the optional survey and QPR simulation. Therefore, this subset served as a close approximation of the total study population.

The participant pool consisted of adults (age 18 and above) with a mean age of 44.69 years (SD = 14.64) and represented diverse professional backgrounds: mental health professionals (*n* = 25, 52% of respondents), education professionals (*n* = 10, 21%), researchers (*n* = 5, 10%), and other professions (*n* = 8, 17%). The mental health professionals were primarily individuals in training or working in mental health-related fields but were not engaged in professional clinical work involving suicide risk assessment. Regarding prior training and experience, only six respondents (13%) reported having received formal, structured training in suicide risk assessment. However, 23 respondents (48%) specifically reported having served as gatekeepers for individuals experiencing suicidal crises.

### Procedure

The 54 simulated conversations analyzed in this study were collected during the time of a single live webinar (online conference) initiated by the authors and offered free of charge to attendees. An initial pool of 65 conversations was collected, with 11 excluded from analysis due to incomplete interaction. Webinar participants were recruited primarily from a social media platform focused on the intersection of GenAI and responsible mental healthcare.

**Figure 1:**
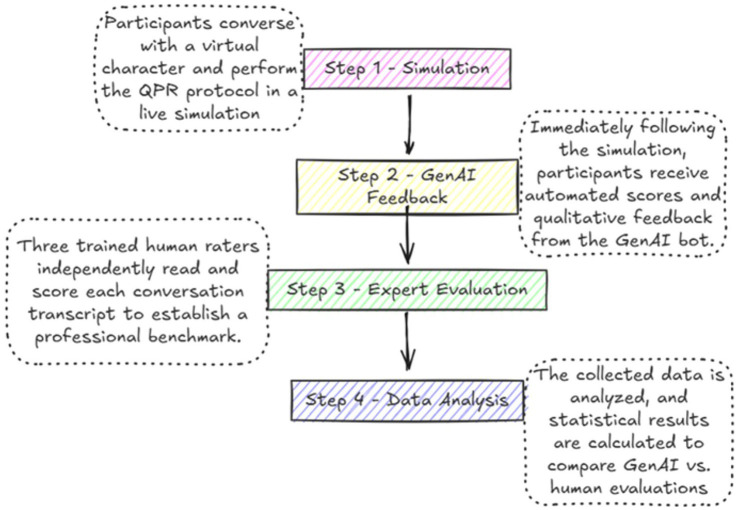
Flow of participant recruitment, simulation procedures, and evaluation in the GenAI-based QPR study.

The webinar, conducted on June 6, 2024 via the Zoom platform, provided a brief introduction to the GenAI QPR simulation tool. Attendees interested in interacting with the simulator were sent a direct link via the Zoom chat function. They were then invited to engage in real-time text-based conversations in which they were to assume the role of mental health gatekeepers. These voluntary interactions were conducted at the participants’ locations and formed the dataset for the current analysis. Each interaction was automatically recorded for research purposes. Prior to the simulation participants were informed that their conversations would be analyzed as part of a research study. Ethical approval for the study was obtained from the university’s Institutional Review Board (Approval Number: 2024–67 YVC EMEK). All procedures complied with strict ethical standards for social science research, including securing informed consent, maintaining anonymity, and ensuring that no personal identification information was collected or stored (See Figure 1).

### GenAI agent used in the current study for QPR simulation and feedback

The agent used in the study was a multistage GenAI-powered simulator designed to provide both a practice environment and immediate formative feedback. This GenAI-based simulator was powered by GPT-4o, governed by a detailed prompt architecture developed by the research team without the use of model fine-tuning or RAG, and deployed via the PMFM AI platform. Each participant’s protocol involved three distinct phases.

#### Phase 1: onboarding and informed consent

Upon accessing the simulation, participants were greeted by the GenAI agent, which then outlined the following scenario: a conversation with Ido, a 29-year-old colleague showing signs of distress following a recent traumatic event. The agent informed participants that their anonymized conversation would be used for research and that upon completion they would receive automated feedback from a secondary GenAI persona called Rotem. Before proceeding and after being assured of anonymity, participants provided the last five digits of their ID number for cross-referencing purposes. The simulation commenced only after participants agreed to these terms.

#### Phase 2: simulation with Ido

After participant consent, GenAI adopted the persona of Ido, a character exhibiting symptoms consistent with post-traumatic stress and depression (see Appendix A for a detailed character profile). GenAI was programmed to adhere to specific behavioral rules to create a realistic training experience, including gradual self-disclosure and constraints, such as not revealing suicidal ideation before the 10th turn of the conversation and not agreeing to seek help before the 20th turn. This design required participants to actively apply their QPR skills.

#### Phase 3: automated feedback with Rotem

When the simulation was complete, GenAI transitioned to the persona of Rotem, a GenAI-based expert clinical psychologist. This agent provided structured, formative, qualitative feedback on the participant’s performance through assessments of Building Rapport, Assessing Risk/Protective Factors, and the three QPR components, citing specific examples from the transcript. See Appendix B for the full instruction prompts of Rotem’s feedback.

After this qualitative review, Rotem assigned a single overall QPR skills score on a 10-point scale based on the following predefined criteria: *Score 9–10 (Exemplary):* Awarded for conversations demonstrating excellent QPR application. These participants built trust quickly, were highly empathetic, thoroughly explored risk and protective factors, skillfully asked about suicide, and facilitated a smooth referral process. *Score 7–8 (Solid):* Assigned for conversations showing good overall QPR skills. These participants were empathetic, identified the most risk factors, and successfully executed the Q, P, and R stages, with only minor areas for refinement. *Score 5–6 (Moderate/Inconsistent):* Assigned for conversations in which QPR competency was present but inconsistent. These participants may have built some rapport but struggled with persuasion or referral or identified only some risk factors. *Score 3–4 (Significant Gaps):* Reserved for interactions with significant deficiencies in QPR skills. Participants struggled to build trust, minimized risk factors, or failed in adequately performing one or more of the core QPR actions. *Score 1–2 (Severe Failures):* Assigned in cases of severe ethical or professional lapses, including dismissive, judgmental, or disrespectful communication; ignoring clear and urgent warning signs; or a complete failure to apply the QPR model.

### Evaluation instruments

To analyze the conversations, both human raters and the main GenAI model assessed each interaction for adherence to the QPR model. Accordingly, each conversation was independently assessed by three human raters with professional backgrounds in psychology. Overall adherence served as a global rating of the conversation’s comprehensive alignment with the QPR model. This item was rated independently on a 10-point scale based on the guiding question, “To what extent does this conversation align with the QPR model components?”

Three independent raters with professional backgrounds in psychology conducted the human evaluations. All raters received formal training in the QPR model, and their rating process was continuously monitored and calibrated in consultation with a senior QPR-certified trainer. To ensure reliability, the expert reviewers sampled and cross-validated the assessments of the three raters throughout the evaluation process.

### Data analysis

The data were analyzed using both descriptive and inferential methods, in line with the study’s three research questions. For RQ1, intraclass correlation coefficients (ICCs) were computed (two-way mixed-effects model, consistency definition) to assess the inter-rater reliability among human raters for each QPR component and for overall adherence. Pearson correlations were then calculated to examine the alignment between GenAI scores and human ratings as well as internal consistency among human raters. For RQ2, repeated-measures ANOVA was used to test whether participant gender influenced adherence scores across both human and GenAI raters. Pearson’s correlations were used to assess the associations between conversation length (number of dialog lines) and adherence scores. For RQ3, a thematic analysis was conducted on three GenAI-rated conversations (low, moderate, and high adherence). Each was examined to assess participants’ performance across QPR components in comparison with GenAI’s corresponding qualitative feedback.

## Results

### RQ1: agreement between human and GenAI ratings

#### Agreement on QPR adherence

Consistency of adherence assessments across evaluators was assessed using intraclass correlation coefficients (ICCs) derived from a two-way mixed-effects model with a consistency definition. The ICCs were calculated for overall adherence (Table 1). The analysis revealed excellent inter-rater reliability among the three human raters for overall adherence to the QPR model, with ICC(3, 4) = 0.897, 95% CI [0.843, 0.935], and *F*(53, 159) = 9.662, *p* < 0.001.

**Table 1:** Inter-rater reliability (ICC) for human feedback score adherence to QPR components (*N* = 54).

Raters compared	Component evaluated	ICC(3,k)	95% CI	F	df	*p*-value
Three human raters and GenAI	Overall Adherence to QPR	0.891	[0.829, 0.933]	9.166	(53, 106)	< 0.001

ICC, Intraclass Correlation Coefficient. CI, Confidence Interval. df, degrees of freedom. All *p*-values are two-tailed.

#### Correlations and agreement between human and GenAI ratings

**Figure 2:**
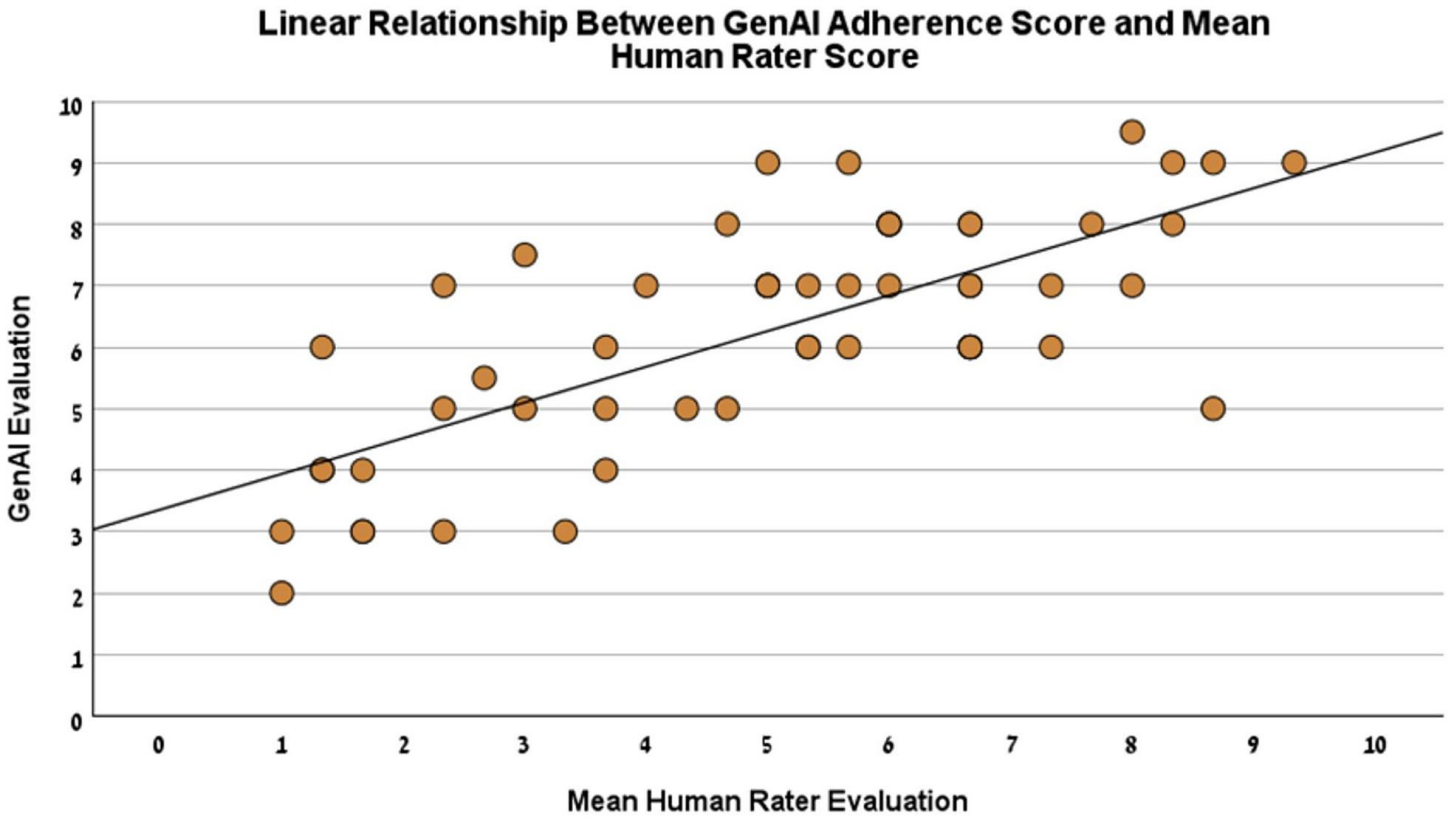
Linear relationship between GenAI and human rater evaluation. This scatter plot illustrates the relationship between the QPR model adherence score awarded by GenAI (Y-axis) and the mean adherence score from the three human raters (X-axis). The plot demonstrates a strong positive linear relationship (*r* = 0.721, *p* < 0.001, *N* = 54), with the solid line representing linear regression.

The criterion validity of the GenAI assessment was evaluated by comparing the scores against the benchmark established by human raters. Pearson’s correlation analysis was conducted between GenAI’s total score and the mean score of the three human raters. The results revealed a strong and statistically significant positive correlation (r(52) = 0.721, *p* < 0.001, see Figure 2). This primary finding indicates a substantial linear relationship between the AI evaluations and the expert human benchmark.

Further analysis showed significant positive correlations between the GenAI’s score and each individual human rater: Tester 1 (*r* = 0.645, *p* < 0.001), Tester 2 (*r* = 0.519, *p* < 0.001), and Tester 3 (*r* = 0.776, *p* < 0.001).

An intraclass correlation coefficient (ICC) was calculated using a two-way mixed-effects model with a consistency definition to provide a more robust measure of agreement. The analysis demonstrated good agreement between the GenAI scores and the mean of the human raters, with an average measure ICC of 0.827 (95% CI [0.702, 0.900]), which was statistically significant, *F*(53, 53) = 5.776, *p* < 0.001.

Despite this strong correlation, consistent variance in scoring was noted. On average, the GenAI rated conversations 1.2 points higher than the human raters’ mean score. This suggests a systematic tendency toward more favorable evaluations in the automated system, which is a critical consideration for calibration.

### RQ2: influence of gender and conversation length

Two analyses were conducted to explore whether participant gender influenced adherence evaluations. First, GenAI scores were compared across gender groups. GenAI adherence scores demonstrated overall alignment with human evaluations, as shown in the previous ICC analysis, suggesting consistent rating patterns across gender groups. A one-way ANOVA was conducted to compare the GenAI scores across the three gender groups, revealing no significant effect of gender [*F* (2, 51) = 2.744, *p* = 0.074]. When examining gender-based differences, the average GenAI score was the highest in conversations with female participants (M = 6.50), slightly lower than that of male participants (M = 6.38), and notably lower in cases in which the participant’s gender was unknown (M = 4.25).

Second, a repeated-measures ANOVA assessed the effect of participant gender on human raters’ adherence scores. The analysis showed no significant main effect of gender [*F*(2, 51) = 2.746, *p* = 0.074] and no significant interaction between gender and rater [*F*(4, 102) = 0.379, *p* = 0.823]. Although the differences were not statistically significant, descriptive means showed that female participants received the highest average scores (M = 5.683), followed by male participants (M = 4.844) and participants of unknown gender (M = 2.917). These findings suggest that GenAI and human raters consistently evaluated QPR adherence across gender groups, with no indication of systematic gender-based bias.

Finally, the influence of conversation length on adherence ratings was examined by calculating the number of dialog lines per simulation and correlating them with human and GenAI scores. The results revealed a significant positive correlation (*r* = 0.27, *p* = 0.030) between the number of lines and average score assigned by human raters. This statistically significant result suggests that longer conversations are modestly associated with higher ratings, possibly due to increased opportunities to demonstrate QPR components, such as empathy or referral. By contrast, no significant correlation was observed between conversation length and GenAI adherence scores, indicating that GenAI evaluations may be less sensitive to interaction length.

### RQ3: qualitative analysis of GenAI-feedback to participants performance

A qualitative review of GenAI’s feedback mechanism was conducted to complement the quantitative analyses and provide deeper insights into its evaluative capabilities. For this purpose, we selected three representative conversations stratified by the final QPR adherence score assigned by the GenAI evaluator personas: low (score = 3–4), moderate (score = 6), and high (score = 8). Tables 2–4 present detailed breakdowns of these conversations. Each table compares examples of participants’ verbatim performance on each QPR component with GenAI’s full corresponding feedback. This comparative analysis illustrates the nature, quality, and nuances of GenAI’s judgment in practice.

**Table 2:** Sample analysis of a conversation with a low adherence score (3/10).

QPR model component	Sample participant quotes	GenAI ‘s full feedback on the component (direct quote from Rotem)
Empathy & risk/protective factor assessment	“That’s not a simple experience.” “I really want to listen, it’s not a burden.” “Do you want to share what thoughts are worrying you?” “How did you know the friend?”	“I saw attempts to understand and show empathy for Ido’s pain, which helped him feel safer to share more. However, you could have placed more emphasis on deepening the connection and on continuous active listening. You did not conduct an in-depth exploration of risk or protective factors during the conversation. You did not delve into Ido’s complex story fully enough to understand his state of mind and the intensity of his pain.”
Direct questioning (Q)	(Not performed)	“You did not directly ask Ido if he is thinking about suicidality or suicide, missing a critical step in the process.”
Persuasion (P)	“It’s truly very painful. Gradually, You can learn to live with the pain.” “This period is very difficult now, gradually it will get easier.”	“You did not explicitly try to persuade Ido to seek professional help. It seemed you instilled some hope, but not in a clear and intentional manner.”
Referral (R)	(Not performed)	“There was no clear suggestion to seek professional help, and no exploration or expansion on the help options available to you for Ido.”
GenAI’s general feedback and final score		“Partial use of QPR skills, with significant errors such as the absence of a direct inquiry about suicidal thoughts, and a lack of clear, intentional attempts at persuasion and offering professional help. Further work is needed to deepen the knowledge and experience in suicide prevention and in building a more empathetic and heartfelt connection. Overall Score: 3/10 There are many things that can be improved, and as with most things-experience is key. Maintain your empathetic approach and strive to understand everything the person in front of you is going through. Do not be afraid to ask directly about suicide and do not forget to offer help from the bottom of your heart. If you have any further questions or would like to discuss the details, I am here for you. Thank you for participating and for your focus on learning and improving your QPR skills.”

**Table 3:** Example analysis of a conversation with a medium GenAI adherence score (6/10).

QPR model component	Participant’s quotes examples	GenAI ‘s full feedback on the component (direct quote from Rotem)
Empathy & risk/protective factor assessment	“I’m sorry you are not sleeping at night.” “What are the most difficult thoughts going through your head?” “Where in your body do you feel it?” “Are you feeling this is the end?”	“You showed empathy when you expressed sorrow for Ido’s condition. However, the start of the conversation was a bit too direct (‘Do you have suicidal thoughts recently?’). It’s better to start with more open-ended questions to allow him to feel comfortable and build trust gradually. You identified the main signs of Ido’s condition, like suicidal thoughts and PTSD symptoms. However, you did not mention the feelings of guilt that can play a central role in Ido’s sense of helplessness.”
Direct questioning (Q)	“Do you have suicidal thoughts recently?” “Have you tried to do anything about it?”	“You asked directly about suicidal thoughts in a good and open manner. This is a critical step in the QPR model, and you deserve praise for that.”
Persuasion (P)	(Not clearly performed; a persuasive element is embedded in the referral)	“When you expressed sorrow and understood his feelings, you managed to create a supportive environment. However, you did not try to encourage him to find hope or remind him of people and resources that could help him.”
Referral (R)	“Would you like to meet with a trauma expert? (*Implied: to help you process what happened*)” “Do you want to try?”	“You recommended seeing a trauma expert, which is the right step. However, it would have been better to inquire more about his current ability to access treatment, and perhaps also offer more immediate options like a hotline or a university psychologist nearby.”
GenAI’s general feedback and final score		“Your QPR practice was good overall, but there is room for improvement in persuasion and referral to professional help. The key points for improvement are: (1) Starting the conversation with more open-ended questions to build trust. (2) Checking his personal capabilities and support systems and offering immediate support if needed. (3) Encouraging hope and building coping mechanisms.” Overall Score: 6/10 “You did a good job identifying Ido’s distress and referring him to professional help, but there was a lack of improvement in the process of persuasion and building hope and support. To reach a higher level of expertise, continue to practice the model and study its components in depth. If you have any further questions or would like to continue practicing, I am here to help. Thank you for your participation and have a good day!”

**Table 4:** Example analysis of a conversation with a high GenAI adherence score (8/10).

QPR model component	Participant’s quotes examples	GenAI’s full feedback on the component (direct quote from Rotem)
Empathy & risk/protective factor assessment	“For me you are important, tell me a little.” “What happens when you try to sleep at night?” “That sounds so painful. Have you ever gone through something similar?” “I understand what you are feeling. I’m right here with you, and I can say that it’s possible to get out of this.	“You demonstrated solid empathetic listening throughout the conversation. Your willingness to assist and your non-judgmental approach helped build trust with Ido, who eventually opened up about his struggles. This rapport was crucial in his willingness to consider seeking help. You successfully identified the key risk factors such as Ido’s feelings of hopelessness, guilt, and contemplation of suicide. By asking directly about his thoughts of suicide, you showed that you were aware of the potential severity of his situation. However, exploring more protective factors, like his family and any previous positive coping strategies, could have been beneficial.”
Direct questioning (Q)	“Do you also think about suicide sometimes?”	“You asked directly about suicidal thoughts with sensitivity and clarity. This showed competency in addressing the issue head-on and assessing the risk without hesitation.”
Persuasion (P)	“I’m right here with you, and I can say that it’s possible to get out of this.” “I know it might be hard for you to believe now, but it’s possible to get out of this and you do not have to be alone in this.” “But of course I want you not to give up on yourself and also to share this with your partner.”	“Your active listening, compassionate responses, and personal anecdotes provided Ido with a sense of hope. You effectively persuaded him to acknowledge his struggles and consider taking steps towards getting help.”
Referral (R)	“Are you willing to see a psychologist? Do you know any good ones?” “What do you think about me connecting you with someone great?” “I’m right here with you, I can even come with you to a psychologist.”	“You excelled in recommending professional support and provided a concrete plan by offering to accompany him to a psychologist. This hands-on approach reinforced your commitment to his well-being and increased the likelihood of him following through.”
GenAI’s general feedback and final score		“Your conversation exhibited strong QPR competencies, displaying empathy, direct questioning, and effective persuasion. By building a foundation of trust and care, you managed to move Ido towards considering professional help, demonstrating key aspects of QPR successfully.” Score: 8/10 “You showed solid QPR skills, built good rapport, and addressed risk factors effectively. Slight improvements could be made in exploring more protective factors and sustaining the conversation about Ido’s feelings before moving towards solutions. Nonetheless, you handled the situation with care and competence.”

This qualitative deep dive highlights several key capabilities of GenAI’s feedback system, demonstrating its significant potential for providing meaningful and scalable feedback in a sensitive and complex domain such as QPR. First, the analysis revealed the accuracy of GenAI’s diagnostic judgment, with GenAI consistently and correctly identifying the presence or absence of core QPR components across all performance levels. Second, the feedback demonstrates considerable depth in that it moved beyond simple scoring to offer integrative, actionable recommendations tailored to specific interactions. Third, GenAI exhibited a remarkable ability to modulate its tone appropriately, sensitively delivering empathic, critical, and professional messages in low-scoring interactions while offering praise and nuanced points for refinement in high-scoring ones. Together, these strengths suggest that GenAI-driven evaluations can provide a foundation for robust, scalable, evidence-based learning.

## Discussion

This study aimed to evaluate the potential of GenAI-generated feedback as a supportive tool for QPR gatekeeper training for suicide prevention. The findings across the three research questions provide preliminary evidence of the reliability and pedagogical value of GenAI while also highlighting important limitations that call for careful calibration and cautious integration into training contexts.

*RQ1: Alignment between GenAI and human raters:* The results revealed a moderate to strong correlation between GenAI adherence scores and the evaluations of trained human raters, along with excellent inter-rater reliability among human experts. This finding is consistent with prior research demonstrating the ability of GenAI-based simulations to provide reliable evaluations in clinical and educational training contexts (20, 21). Nevertheless, GenAI systematically produced scores that were, on average, 1.2 points higher than those assigned by human raters. Such a pattern corresponds with the “generosity bias” or “sycophantic bias” observed in generative models (25), which may stem from a preference for positive reinforcement and an avoidance of negative feedback unless explicitly prompted (26). Another explanation is that GenAI appears to rely primarily on explicit verbal markers of QPR adherence, whereas human rates also attend to more implicit relational features, such as timing, coherence, and affective nuance (27). These findings underscore the potential of GenAI as a formative assessment tool but also highlight the need for calibration before it can be employed in high-stakes or summative evaluations (20, 28).

*RQ2: Influence of gender and conversation length*: With respect to the second research question, no statistically significant differences were found in adherence scores across gender groups, whether rated by the GenAI or by human raters. This outcome supports previous calls to empirically test, rather than assume, the existence of gender-based biases in AI-mediated communication assessments (23). Although participants of unknown gender received lower scores, a qualitative review indicated that their conversations were shorter and less developed, suggesting brevity, rather than gender ambiguity, as a likely explanation.

The analyses further showed a small, yet significant, positive correlation between conversation length and adherence scores assigned by human raters but not by GenAI. In other words, human evaluators tended to reward longer conversations that provided greater opportunities to demonstrate key QPR components such as persuasion or referral. In contrast, the GenAI scores remained relatively stable, regardless of length. This divergence can be interpreted through Corbin et al.’s (29) recognition-based framework: Human raters are sensitive to relational cues that accumulate across successive turns, whereas GenAI, operating in a checklist-like “extra-recognitive” mode, focuses on the surface-level presence of specific phrases rather than the dialogical process.

*RQ3: Qualitative analysis of GenAI feedback*: The qualitative analysis of three representative conversations reinforced the quantitative findings and illustrated the pedagogical strengths of GenAI feedback. The system consistently and accurately identified the presence or absence of QPR components, offered tailored suggestions for improvement, and appropriately modulated its tone according to performance level, providing empathic yet critical messages for low-performing participants and praise with nuanced recommendations for high performers. These results align with those of previous studies indicating that GenAI-based training can enhance emotional realism, self-reflection, and skill acquisition in sensitive domains (18, 19). At the same time, reliance on text-based input constitutes a clear limitation: GenAI cannot interpret nonverbal cues such as tone of voice, facial expressions, or hesitation, which often play a crucial role in crisis conversations (30). As such, while GenAI provides structured and consistent formative feedback, human facilitation remains essential in contexts in which emotional nuances and experiential learning are critical (17, 31–33).

### AI-human interaction considerations

Beyond these technical constraints, it is important to recognize that the nature of AI-mediated evaluation fundamentally differs from human mentorship in ways that extend beyond the specific capabilities measured in this study. While GenAI can provide a consistent and scalable assessment of observable QPR components, the pedagogical relationship between trainees and evaluators —characterized by trust, emotional safety, and collaborative meaning-making—operates differently in human-AI versus human-human interactions (27). This distinction does not diminish GenAI’s value as a training tool but rather underscores the need to carefully consider how automated feedback is positioned within broader learning ecosystems and to maintain awareness of the qualitative differences between technological scalability and relational authenticity in formative assessment.

### Practical implications

The findings of this study suggest several directions for integrating GenAI-based simulations into suicide-prevention training. GenAI feedback can serve as a valuable supplement to traditional role-play and instructor-led methods by providing scalable and individualized opportunities for deliberate practice. Training programs in schools, universities, and healthcare systems could incorporate GenAI modules between in-person sessions, allowing trainees to rehearse skills repeatedly in a safe, low-stakes environment and receive immediate and structured feedback. This integration may help address the long-recognized limitations of conventional QPR training, such as unequal participation opportunities and reliance on self-reported confidence. Simultaneously, the observed “generosity bias” highlights the importance of implementing GenAI feedback under human supervision. Educators and supervisors can use GenAI-generated scores and qualitative reports as formative scaffolds while retaining responsibility for the final evaluations. Embedding GenAI feedback in blended learning models in which automated assessments are complemented by expert debriefing may maximize both scalability and pedagogical rigor while reducing the risk of over-reliance on surface-level cues, thus ensuring that trainees receive guidance on the deeper relational dynamics that are central to suicide prevention conversations.

Moreover, it is critical to emphasize that this study’s focus was on training suicide prevention gatekeepers—the essential first line of response—rather than replacing professional mental health treatment. The purpose of GenAI-based simulation is to address the urgent need for scalable and accessible training that can expand the capacity of frontline responders to identify and refer at-risk individuals to appropriate professional care. GenAI tools are designed to augment the irreplaceable expertise of trained human clinicians. The ultimate goal is to enhance the preparedness and confidence of human gatekeepers, who remain an essential bridge between at-risk individuals and professional mental health services.

### Limitations

This study had several limitations that may provide directions for future research. First, the central limitation was the sample size, which was based on a convenience sample of 54 participants recruited from a single live webinar. While we are committed to advancing innovation in AI-based mental health training, we believe it is essential to do so with caution and responsibility. Therefore, this study was intentionally designed as an initial proof-of-concept to establish preliminary feasibility and provide foundational evidence for GenAI’s potential as a reliable assessment tool rather than to achieve broad generalizability. Although this sample size exceeds the minimum thresholds recommended for ICC-based inter-rater reliability studies (typically 30–50 participants), it limits the generalizability of our findings. The current findings should be interpreted as a necessary first step that warrants further replication and extension. Future research should include larger and more diverse samples and multi-site studies to establish broader validity and comparative effectiveness across different populations and training contexts.

An additional demographic limitation concerns the information provided to AI. In the current study, no demographic information beyond gender was provided to the AI system, both to minimize potential biases and protect participant privacy. Future research should examine how providing demographic information to AI, such as culture, socioeconomic status, and educational background, influences its feedback quality and communication style. This is critical for determining whether GenAI tools maintain fairness across diverse users or exhibit systematic biases.

Second, the validation benchmark was based on only three human raters. While this meets the standard requirements for inter-rater reliability assessment and yielded excellent agreement (ICC = 0.897), with all raters receiving formal QPR training and continuous calibration by a senior certified trainer, expanding the evaluator panel would strengthen the validation process. Future studies should include a broader range of expert evaluators with diverse cultural backgrounds and gender representation to establish a more robust gold standard. Such diversity would enable an examination of whether AI feedback maintains consistent reliability across evaluator backgrounds or exhibits systematic biases.

Third, the study design did not include control groups. We did not use a control group (e.g., traditional role-play), which means that, although we validated GenAI’s assessment reliability, we cannot make claims about the comparative effectiveness of this training method. Furthermore, our analysis focused on a single, summative, one-way AI feedback model provided at the conclusion of the simulation. We did not compare the effectiveness of this AI feedback against qualitative feedback provided by a human expert, nor did we explore other AI feedback modalities, such ad dialogic feedback between participants and AI, real time feedback during the simulation and a combination between AI and human feedback (29, 34, 35). Future research must be designed to thoroughly examine the effectiveness and reliability of various feedback models and their value in comparison to traditional human feedback.

Fourth, we only compared GenAI’s global adherence score and did not analyze the QPR component-level scores. Future research should examine the ability of GenAI to reliably rate individual QPR subcomponents, enabling more nuanced comparisons with human judgment.

Fifth, while our qualitative review provides illustrative examples, a systematic content analysis of GenAI’s feedback is needed to rigorously map its strengths, weaknesses, and potential biases.

Finally, future work should explore GenAI’s performance in richer voice-based modalities and directly investigate the user’s subjective experience of receiving automated feedback, possibly through “human-in-the-loop” models that synthesize technological and human strengths.

## Conclusion

This study provides foundational evidence that Generative AI can serve as a reliable and scalable tool both for training and for evaluating life-saving skills in suicide prevention. The study’s findings represent a critical step toward moving from GenAI as a simulator to GenAI as a valid assessor. The findings highlight that when GenAI-generated feedback is carefully designed and benchmarked, it has the potential to complement existing approaches by offering accessible, consistent, and individualized assessments in real time. While the path toward a fully autonomous GenAI evaluator requires a critical and cautious approach to address the current limitations, the results of this study demonstrate the significant potential of harnessing GenAI to strengthen the competence and confidence of gatekeepers. In this way, GenAI-powered training can help overcome the traditional barriers of time, geography, and language, providing wider access to practice, feedback, and skill development in suicide prevention.

## Data Availability

The raw data supporting the conclusions of this article will be made available by the authors, without undue reservation.

## References

[1] Mann JJ Michel CA Auerbach RP. Improving suicide prevention through evidence-based strategies: a systematic review. Am J Psychiatry. (2021) 178:611–24. doi: 10.1176/appi.ajp.2020.20060864, 33596680 PMC9092896

[2] Lovero KL Dos PF Come AX Wainberg ML Oquendo MA. Suicide in global mental health. Curr Psychiatry Rep. (2023) 25:255–62. doi: 10.1007/s11920-023-01423-x, 37178317 PMC10182355

[3] Burnette C Rajeev Ramchand Ayer L. Gatekeeper training for suicide prevention: a theoretical model and review of the empirical literature. Rand Health Q. (2015) 5:16–6. doi: 10.48550/arXiv.2505.13480 PMC5158249 28083369

[4] Holmes G Clacy A Hamilton A Kõlves K. Do positive gatekeeper training outcomes predict gatekeeper intervention Behaviours? Arch Suicide Res. (2025) 29:1043–56. doi: 10.1080/13811118.2025.2469882, 39987574

[5] Kingi-Uluave D Taufa N Tuesday R Cargo T Stasiak K Merry S . A review of systematic reviews: gatekeeper training for suicide prevention with a focus on effectiveness and findings. Arch Suicide Res. (2024) 29:329–46. doi: 10.1080/13811118.2024.2358411, 38884349

[6] Bonnin R Gralnik LM Rothe E Obeso V von Harscher H Shoua-Desmarais N . Overcoming stigma: a novel curriculum for teaching medical students about suicide. Acad Psychiatry. (2021) 45:751–6. doi: 10.1007/s40596-021-01485-0, 34080134

[7] Burgues M Goujet R Zaraik J. Learning soft skills with an ai-based simulation role-play: A literature review. Eduleern Proceed. (2024) 6285–6293. doi: 10.21125/edulearn.2024.1484

[8] Aldrich RS Wilde J Miller E. The effectiveness of QPR suicide prevention training. Health Educ J. (2018) 77:964–77. doi: 10.1177/0017896918786009

[9] Gryglewicz K Garrison CMT Childs KK Labouliere CD Karver MS. Examining individual and service delivery context variables and their association with the effectiveness of QPR suicide prevention gatekeeper training. Adm Policy Ment Health Ment Health Serv Res. (2024) 51:47–59. doi: 10.1007/s10488-023-01308-4, 37861855

[10] Quinnett P. QPR gatekeeper training for suicide prevention: the model, theory and research. Spokane, WA, USA: QPR Institute (2007) 4.

[11] Bartgis J Albright G. Online role-play simulations with emotionally responsive avatars for the early detection of native youth psychological distress, including depression and suicidal ideation. Am Indian Alsk Native Ment Health Res. (2016) 23:1–27. doi: 10.5820/aian.2302.2016.1, 27115130

[12] Cross WF Seaburn D Gibbs D Schmeelk-Cone K White AM Caine ED. Does practice make perfect? A randomized control trial of behavioral rehearsal on suicide prevention gatekeeper skills. J Prim Prev. (2011) 32:195–211. doi: 10.1007/s10935-011-0250-z, 21814869 PMC3249637

[13] McGaghie WC Siddall VJ Mazmanian PE Myers J. Lessons for continuing medical education from simulation research in undergraduate and graduate medical education. Chest. (2009) 135:62S–8S. doi: 10.1378/chest.08-2521, 19265078

[14] Maicher KR Zimmerman L Wilcox B Liston B Cronau H Macerollo A . Using virtual standardized patients to accurately assess information gathering skills in medical students. Med Teach. (2019) 41:1053–9. doi: 10.1080/0142159x.2019.1616683, 31230496

[15] Plöderl M Fartacek C Kapitany T Schrittwieser U Niederkrotenthaler T. Effects of gatekeeper trainings from the Austrian national suicide prevention program. Front Psychiatry. (2023) 14. doi: 10.3389/fpsyt.2023.1118319, 37547202 PMC10397513

[16] Lauderdale SA Schmitt R Wuckovich B Dalal N Desai H Tomlinson S. Effectiveness of generative AI-large language models’ recognition of veteran suicide risk: a comparison with human mental health providers using a risk stratification model. Front Psychiatry. (2025) 16. doi: 10.3389/fpsyt.2025.1544951, 40248601 PMC12003356

[17] Levkovich I. Evaluating diagnostic accuracy and treatment efficacy in mental health: a comparative analysis of large language model tools and mental health professionals. European J Investigation Health Psychol Educ. (2025) 15:9–9. doi: 10.3390/ejihpe15010009, 39852192 PMC11765082

[18] Haber Y Hadar Shoval D Levkovich I Yinon D Gigi K Pen O . The externalization of internal experiences in psychotherapy through generative artificial intelligence: a theoretical, clinical, and ethical analysis. Front Digital Health. (2025) 7:1512273. doi: 10.3389/fdgth.2025.1512273, 39968063 PMC11832678

[19] Elyoseph Z Levkovich I Haber Y Levi-Belz Y. Using GenAI to train mental health professionals in suicide risk assessment. J Clin Psychiatry. (2025) 86. doi: 10.4088/jcp.24m15525 40608482

[20] Stamer T Steinhäuser J Flägel K. Artificial intelligence supporting the training of communication skills in the education of health care professions: scoping review. J Med Internet Res. (2023) 25:e43311. doi: 10.2196/43311, 37335593 PMC10337453

[21] Share P Pender J. Role-playing the future: A simulation-based exploration of AI in social work. Ubiquity proceed. (2024) 27:149:10.5334/uproc.149

[22] Levkovich I Haber Y Levi-Belz Y Elyoseph Z. A step toward the future? Evaluating GenAI QPR simulation training for mental health gatekeepers. Front Med. (2025) 12. doi: 10.3389/fmed.2025.1599900, 40568211 PMC12187690

[23] Nadeem A Abedin B Marber P. Gender bias in AI-based decision-making systems: A systematic literature review. Australasian Journal of Information Systems. (2022) 26:1–34. doi: 10.3127/ajis.v26i0.3835

[24] Patil A Tao S Gedhu A. Evaluating reasoning LLMs for suicide screening with the Columbia-suicide severity rating scale. arXiv preprint arXiv. (2025). doi: 10.48550/arXiv.2505.13480

[25] Michels S. Teaching (with) artificial intelligence: the next twenty years. J Polit Sci Educ. (2023) 20:510–21. doi: 10.1080/15512169.2023.2266848

[26] Sharma M. Tong M. Korbak T. Duvenaud D. Askell A. Bowman S. R. (2024). “Towards understanding sycophancy in language models.” In Proceedings of the International Conference on Learning Representations (ICLR 2024). OpenReview.

[27] Nygren T Samuelsson M Hansson PO Efimova E Bachelder S. AI versus human feedback in mixed reality simulations: comparing LLM and expert mentoring in preservice teacher education on controversial issues. Int J Artif Intell Educ. (2025) 35:2856–88. doi: 10.1007/s40593-025-00484-8

[28] Lim T Gottipati S Cheong ML. Ethical considerations for artificial intelligence in educational assessments In: Creative AI tools and ethical implications in teaching and learning: IGI Global, Eds. J. Keengwe (2023). 154–170. doi: 10.4018/979-8-3693-0205-7.ch008

[29] Corbin T Tai J Flenady G. Understanding the place and value of GenAI feedback: a recognition-based framework. Assess Eval High Educ. (2025) 50:718–31. doi: 10.1080/02602938.2025.2459641, 41307611

[30] Haynal-Reymond V Jonsson GK Magnusson MS. Non-verbal communication in doctor-suicidal patient interview In: Anolli L Duncan S Jr Magnusson MS Riva G, editors. The hidden structure of interaction: From neurons to culture patterns. Amsterdam: IOS Press. (2005). 141–8.

[31] Maurya RK. Using AI based chatbot ChatGPT for practicing counseling skills through role-play. J Creat Ment Health. (2023) 19:513–28. doi: 10.1080/15401383.2023.2297857

[32] Meinlschmidt G Koc S Boerner E Tegethoff M Simacek T Schirmer L . Enhancing professional communication training in higher education through artificial intelligence (AI)-integrated exercises: study protocol for a randomised controlled trial. BMC Med Educ. (2025) 25:804. doi: 10.1186/s12909-025-07307-3, 40448046 PMC12123891

[33] Turner S Harder N Martin D Gillman L. Psychological safety in simulation: perspectives of nursing students and faculty. Nurse Educ Today. (2023) 122:105712. doi: 10.1016/j.nedt.2023.105712, 36669303

[34] Louie R Orney I Pacheco J Shah R Brunskill E Yang D. Can LLM-simulated practice and feedback upskill human counselors? A Randomized Study with 90+ Novice Counselors (2025). doi: 10.48550/arXiv.2505.02428

[35] Lin I. Sharma A. Rytting C. Miner A. Suh J. Althoff T., (2024). IMBUE: Improving interpersonal effectiveness through simulation and just-in-time feedback with human-language model interaction. In Proceedings of the 62nd Annual Meeting of the Association for Computational Linguistics (Volume 1: Long Papers), pages 810–840, Bangkok, Thailand. Association for Computational Linguistics. Available online. doi: 10.18653/v1/2024.acl-long.47

[36] Elyoseph Z Gur T Haber Y Simon T Angert T Navon Y . An ethical perspective on the democratization of mental health with generative artificial intelligence. JMIR Ment Health. (2024) 11:e58011. doi: 10.2196/58011 39417792 PMC11500620

[37] Lee SS Moore RL. Harnessing generative AI (GenAI) for automated feedback in higher education: A systematic review. Online Learning. (2024) 28:82–104. doi: 10.24059/olj.v28i3.4593

[38] Corbin T Tai J Flenady G. Understanding the place and value of GenAI feedback: a recognition-based framework. (2025). doi: 10.1080/02602938.2025.2459641

